# Estimation of wheat tiller density using remote sensing data and machine learning methods

**DOI:** 10.3389/fpls.2022.1075856

**Published:** 2022-12-21

**Authors:** Jinkang Hu, Bing Zhang, Dailiang Peng, Ruyi Yu, Yao Liu, Chenchao Xiao, Cunjun Li, Tao Dong, Moren Fang, Huichun Ye, Wenjiang Huang, Binbin Lin, Mengmeng Wang, Enhui Cheng, Songlin Yang

**Affiliations:** ^1^ Key Laboratory of Digital Earth Science, Aerospace Information Research Institute, Chinese Academy of Sciences, Beijing, China; ^2^ College of Resource and Environment, University of Chinese Academy of Sciences, Beijing, China; ^3^ International Research Center of Big Data for Sustainable Development Goals, Beijing, China; ^4^ Land Satellite Remote Sensing Application Center, Ministry of Natural Resources of China, Beijing, China; ^5^ Information Technology Research Center, Beijing Academy of Agriculture and Forestry Sciences, Beijing, China; ^6^ Aerospace ShuWei High Tech. Co., Ltd., Beijing, China; ^7^ Beijing Azup Scientific Co., Ltd., Beijing, China; ^8^ Department of Geography, Texas A&M University, TX, United States; ^9^ School of Geography and Information Engineering, China University of Geosciences (Wuhan), Wuhan, China

**Keywords:** winter wheat, tiller density, UAV hyperspectral, vegetation index, random forest, gradient boosted regression trees

## Abstract

The tiller density is a key agronomic trait of winter wheat that is essential to field management and yield estimation. The traditional method of obtaining the wheat tiller density is based on manual counting, which is inefficient and error prone. In this study, we established machine learning models to estimate the wheat tiller density in the field using hyperspectral and multispectral remote sensing data. The results showed that the vegetation indices related to vegetation cover and leaf area index are more suitable for tiller density estimation. The optimal mean relative error for hyperspectral data was 5.46%, indicating that the results were more accurate than those for multispectral data, which had a mean relative error of 7.71%. The gradient boosted regression tree (GBRT) and random forest (RF) methods gave the best estimation accuracy when the number of samples was less than around 140 and greater than around 140, respectively. The results of this study support the extension of the tested methods to the large-scale monitoring of tiller density based on remote sensing data.

## 1 Introduction

Wheat is one of the world’s most important food crops and provides food for more than half of the world’s population (Grassini et al., 2013; Blackie, 2016). With the world population expected to reach 9 billion by 2050, demand for wheat is expected to increase by 60%–110% (Godfray et al., 2010; Tilman et al., 2011; Ray et al., 2013). To meet this demand, annual wheat yield increases must rise from the current value of less than 1% to at least 1.6% (Tilman et al., 2011; Ray et al., 2013). Wheat’s yield potential depends on the tiller density at the tillering stage (Elsayed et al., 2018) and, under normal or high-density sowing scenarios, tillers produced in winter wheat from fall until the beginning of January of the following year constitute more than 87% of the final yield (Tilley et al., 2019). The tiller density is also closely related to the nitrogen status of winter wheat (Elsayed et al., 2018). Therefore, accurate, efficient, and real-time knowledge of the tiller density during the tillering stage of winter wheat is important for improving nitrogen fertilization management, obtaining an optimal seed yield, and implementing sustainable agricultural practices (Cheng, 2020).

The tiller density refers to the number of tillers of winter wheat contained in a unit area (e.g., 1 m^2^). Currently, the most common method for measuring the tiller density is manual counting, which is extremely time-consuming and inefficient, limited by human error, and lacking in timeliness and accuracy (Scotford and Miller, 2004). Remote sensing provides an alternative method due to its ability to provide quantitative biophysical parameter data for vegetation in a non-contact and non-destructive manner (Zenkl et al., 2021). Remote sensing estimation methods of tiller density in the literature can be generally classified into two types: (1) image segmentation models and (2) spectral feature models. Both 2D and 3D image segmentation models are available: the 2D approaches are based on 2D RGB images taken by handheld cameras or unmanned aerial vehicles (UAVs) and make use of methods such as manually designed features (Liu et al., 2016; Liu et al., 2017; Liu et al., 2018) or machine learning (Jin et al., 2017) to segment leaf image elements so that the tiller density can be estimated under field conditions in sample plots. These methods require a high image resolution (ground sampling distance< 0.5 mm). In the 3D approaches, point clouds of wheat are obtained with the help of remote sensing techniques such as LIDAR, and the tiller number is estimated by clustering (Roth et al., 2020; Fang et al., 2020). This can be severely affected by wind and shading between wheat leaves and cause the tiller number to be underestimated (Fang et al., 2020). Spectral characterization models, in contrast, establish a regression between the tiller density and vegetation indices (VIs) to estimate the tiller density (Flowers et al., 2001; Flowers et al., 2003; Scotford and Miller, 2004; Phillips et al., 2004; Wu et al., 2011; Wu et al., 2022). Most regression models use linear, a few use non-linear ones such as exponential regression. Results show that VIs are reliable indicators for estimating the wheat tiller density in the field; but the relative error was above 20% and could not meet the 10% accuracy required for the application (Liu et al., 2017).

Most current studies of wheat tiller density or tiller number are based on RGB images acquired on the ground or using UAVs; the tiller density is then estimated using image segmentation, which constitutes a source of point data and cannot be used to estimate the tiller density of the plot as a whole; however, it cannot accurately reflect the spatial variation in the density within and between plots. Details of this spatial variation can only be visualized by using a spatial interpolation algorithm and the values of the wheat tiller density that have been obtained, which are subject to errors caused by spatial heterogeneity. In addition, in the case of larger areas, there are difficulties in obtaining UAV data. Developments in high-resolution satellite remote sensing are helping this situation: in particular, spectral feature models can be used to estimate the wheat tiller density on a pixel-by-pixel basis. Therefore, the actual number of tillers of winter wheat can be estimated by using high-resolution satellite images acquired in late fall and early winter based on a small number of measured tillers (Miller and Adkins, 2021); maps showing the spatial distribution of the tiller density can then be obtained.

Traditional methods of inverting crop physicochemical parameters are mainly based on parametric regression of a single vegetation index (VI) as a variable (Verrelst et al., 2015), which is widely used to estimate crop parameters and monitor crop conditions (Bahrami et al., 2021), is used to establish regression relationships. Such methods tend to be very sensitive to noise (Danner et al., 2021) and are suitable for estimating equations corresponding to different linear or exponential relationships (Liang et al., 2015). However, complex and strongly nonlinear relationships exist between biophysical and biochemical parameters and reflectance spectra that cannot be accurately simulated by these parametric models (Liang et al., 2015); also, these models cannot be transferred to other sites with different vegetation or applied to data acquired using other types of sensors or under different conditions (Lu and He, 2019). However, nonparametric linear and nonlinear regression methods have been developed to overcome these deficiencies. In particular, machine learning (ML) regression algorithms have evolved rapidly in recent decades due to their ability to mine and understand information deep within datasets and have been shown to reliably solve nonlinear problems (Camps-Valls et al., 2018). Because of their ability to obtain crop physical and chemical parameters and satellite reflectances, nonlinear modeling of the relationship between physicochemical parameters and satellite reflectance spectra is increasingly applied in combination with remote sensing techniques for crop growth monitoring (Rehman et al., 2019; Zhang et al., 2019; Zha et al., 2020; Machwitz et al., 2021). It is common practice to extract multiple vegetation indices with different effects from spectral information and filter the most relevant vegetation indices to the target physicochemical parameters by using feature engineering or feature selection (Danner et al., 2021) as the input to train machine learning regression models (e.g., support vector regression (SVR), Gaussian process regression (GPR), random forest (RF), and gradient boosted regression trees (GBRT)). The model with the highest estimation accuracy is then obtained by optimizing and adjusting the model hyperparameters and the cross-validation results. Generally, the number of filtered features does not exceed 15%–20% of the total number of field measurement samples, which means that the risk of overfitting can largely be avoided (Thenkabail et al., 2000). Machine learning methods have evolved as reliable methods of learning nonlinear relationships because they require less parameterization, are implemented at various spatial and temporal scales, and are more robust and covariant to noisy features, small training sizes, and large numbers of dimensions (Verrelst et al., 2012; Liang et al., 2015; Houborg and McCabe, 2018). These methods have been widely used for estimating various biophysical parameters such as the leaf area index (Duan et al., 2019; Tao et al., 2020), vegetation cover (Niu et al., 2021; Yu et al., 2021), biomass (Yue et al., 2019; Tao et al., 2020), Canopy chlorophyll content (Jiao et al., 2021) and the leaf tilth distribution (Zou et al., 2022). However, few studies have been conducted to estimate the tiller density of winter wheat.

Therefore, in this study, models for estimating the tiller density based on multiple vegetation indices using machine learning methods were established. Results with higher accuracy than those obtained in previous research were achieved. Corresponding spatial distribution maps based on different types of remote sensing data (including hyperspectral and multispectral data) were also obtained. It was verified that a machine learning model for estimating the winter wheat tiller density based only on plot-scale samples can be extended to the county scale. In this paper, the use of digital imagery instead of manual counts to determine tiller density as a way of obtaining ground truth data that is less time-consuming and laborious is considered.

## 2 Materials and methods

### 2.1 Field experiments, measurements, and data processing

**Figure 1:**
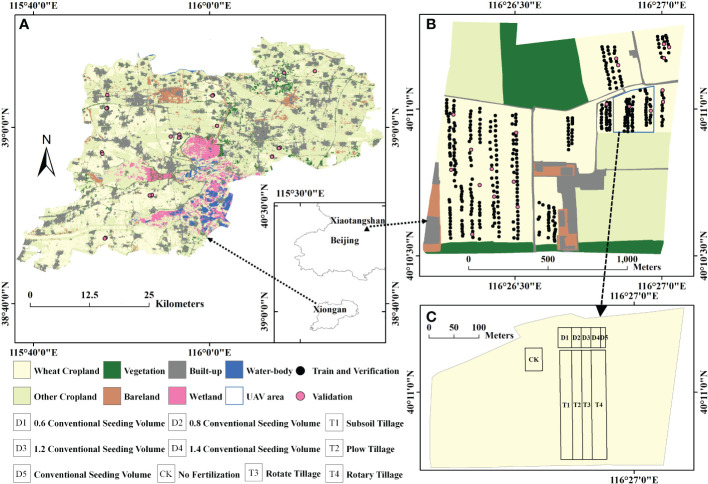
Geographical location of the study sites: the experiments were conducted at **(A)** Xiongan in November 2020, **(B)** Xiaotangshan in November and early December 2020, and **(C)** different application scenario settings at Xiaotangshan.

The ground experiments on which this study was based were conducted at two sites near Beijing, China (**Figure 1**): the Xiaotangshan National Precision Agriculture Research Center (40.10°N, 116.26°E) and Xiongan (38°43′–39°10′N, 115°38′–116°20′E).

The experiments included making the following observations.

① The tiller density was measured by manually counting the number of tillers in a 0.5 m × 0.5 m area around each sampling point. Vertical digital photographs of the same areas were also taken at a 1:1 scale, corresponding to the ground dimensions of 0.5 m × 0.5 m. These photographs were used for training the deep-learning model that was to be used to extract the tiller density from the photographs.
② Hyperspectral data were acquired using a Cubert S185 image hyperspectral sensor carried by a DJI M300 UAV at an altitude of 40 m on November 23, 2020, at the Xiaotangshan study site. (The area over which these images were acquired is shown as the blue box in **Figure 1B**). The data were processed to give the ground reflectance in a total of 125 bands within the wavelength range 450–950 nm with a sampling interval of 4 nm. The ground sample distance (GSD) was resampled from 1 cm to 0.5 m to correspond to the imaged area using the nearest neighbor method.
③ The multispectral data used in this study consisted of Sentinel-2 A/B L1C-level satellite data that covered the study areas shown in **Figure 1**. These data were downloaded from the European Space Agency website (https://scihub.copernicus.eu/dhus/#/home). The Level-2A bottom-of-atmosphere (BOA) reflectance product corresponding to four bands (bands 2, 3, 4, and 8) in the visible and near-infrared range was then obtained by applying the Sen2cor atmospheric correction module provided by ESA to correct for the effects of the atmosphere. For Xiongan, one scene of data from November 2020 was obtained; for Xiaotangshan, four scenes from the period from November to December 2020 were obtained. Further details of the experiments that were carried out at the two study sites are given in **Table 1**. Mid-to-late November and December were chosen for the experiment because winter wheat fertility had already entered the overwintering season at Xiaotangshan and Xiongan, tillering had ceased, and tiller density was almost unchanged during the growth period. The satellite data were also chosen at this time to correspond with the ground experiment time.

**Table 1:** Summary of the experiments performed at Xiaotangshan and Xiongan.

Experiment no.	Month(s) and year	Experiment details
Xiaotangshan study site: training, verification, and validation		
1	Nov 2020	Within the area corresponding to the blue box in **Figure 1B**, observations were made using the ground-based S185 UAV hyperspectral sensor. These observations were made on the same date that the Sentinel-2 satellite data were acquired (November 23). Manually measured tiller density values were collected from 60 points; digital photographs of the same points were taken. (50 of these points were randomly selected for subsequent use as training points; 10 points were used for verifying the accuracy of the estimation model). Ten different application scenarios were set up based on different seeding volumes, different tillage methods, and different fertilization conditions to simulate the tiller density under different scenarios, with specific settings referring to the notes in the lower left corner of **Figure 1** and spatial distribution as shown in **Figure 1C**.
2	Nov and Dec 2020	Within the area of the winter wheat crop marked in **Figure 1B**, the tiller density value was measured at more than 400 points (including the 60 points from experiment 1). Digital photographs were taken of the same points. These data were used for training and verifying the tiller density estimation model for the entire Xiaotangshan wheat growing area; data from 20 points were used for validating subsequent observations. Four Sentinel-2 scenes were acquired during the period of these field measurements (November 16 to December 10). (Note: The field measurements were carried out four separate times to coincide with the satellite transits, but due to weather conditions, the field experiments could not be performed at the same time as the Sentinel-2 transits; however, the time difference was not more than two days on any occasion.)
Xiongan study site: validation		
3	Nov 2020	Within the area of the winter wheat crop area marked in **Figure 1A**, manual measurements of the tiller density were made at 23 points (the pink points in **Figure 1A**) during the period November 7 to November 9. These data were subsequently used for verifying the accuracy of the extended model that was developed in experiment 2. Sentinel-2 satellite data from November 8 were also acquired to coincide with the time of this experiment.

Before constructing the estimation model based on the vegetation indices and tiller density values, 12 vegetation indices (**Table 2**) were first considered. These indices were based on the vegetation structure (e.g., the leaf area index, canopy depression, green biomass, or species) or on biochemical parameters (e.g., chlorophyll or other pigments and nitrogen), which were calculated based on the remote sensing data obtained in the previous treatments. The 12 vegetation indices chosen were all broad-band vegetation indices with no hyperspectral vegetation indices to compare the accuracy of hyperspectral and multispectral data results. The tiller density estimation model was constructed using the red, green, blue, and near-infrared bands of the hyperspectral data that had been shown to have the highest correlation with the tiller density. Calculations for **Table 2**’s vegetation indices utilized hyperspectral data at wavelengths of 458 nm, 492 nm, 750 nm, and 740 nm, respectively.

**Table 2:** Summary of vegetation indexes selected for use in this study.

Vegetation Index	Definition	Features	Reference
Vegetation structure			
**DVI** (Difference Vegetation Index)	*ρ*_*nir*_−*ρ*_*red*_	Estimated vegetation leaf area index	Clevers, 1986
**EVI** (Enhanced Vegetation Index)	2.5×(*ρ*_*nir*_−*ρ*_*red*_)/(*ρ*_*nir*_+6×*ρ*_*red*_−7.5×*ρ*_*blue*_+1)	Capable of improving the sensitivity of estimates of the vegetation leaf area index, biomass, and water content in areas with high biomass	Huete et al., 2002
**MSR** (Modified Red-Edge Simple Ratio Vegetation Index)	$\frac{\frac{\rho_{nir}}{\rho_{red}}-1}{\sqrt{\left(\frac{\rho_{nir}}{\rho_{red}}+1\right)}}$	Linear correlation with vegetation parameters is higher than for the RDVI	Chen, 1996; Haboudane et al., 2004
**MTVI** (Modified Triangular Vegetation Index)	1.2×(1.2×(*ρ*_*nir*_−*ρ*_*green*_)−2.5×(*ρ*_*red*_−*ρ*_*green*_)	Sensitive to the leaf area index; suitable for leaf area index estimation	Haboudane et al., 2004
**NDVI** (Normalized Difference Vegetation Index)	$\frac{\rho_{nir}-\rho_{red}}{\rho_{nir}+\rho_{red}}$	Better response to changes in green biomass; gives improved results for medium- and low-density vegetation	Rouse et al., 1974
**NGRDI** (Normalized Green Red Difference Vegetation Index)	$\frac{\rho_{green}-\rho_{red}}{\rho_{green}+\rho_{red}}$	Minimizes atmospheric effects on estimates of green vegetation	Gitelson et al., 2002
**SR** (Simple Ratio Vegetation Index)	$\frac{\rho_{nir}}{\rho_{red}}$	Similar to the NDVI	Jordan, 1969
**OSAVI** (Optimized Soil-Adjusted Vegetation Index)	$\frac{\left(1+0.16\right)\left(\rho_{nir}-\rho_{red}\right)}{\rho_{nir}+\rho_{red}+0.16}$	More sensitive than the SAVI at the canopy scale and more suitable for agricultural applications	Rondeaux et al., 1996
**RGD** (Red Green Difference Vegetation Index)	*ρ*_*red*_−*ρ*_*green*_	Can be used for estimating vegetation cover	Sanjerehei, 2014
**WDRVI** (Wide Dynamic Range Vegetation Index)	$\frac{0.1\times \rho_{nir}-\rho_{red}}{0.1\times \rho_{nir}+\rho_{red}}$	Capable of providing better estimation results for the leaf area index, biomass, and vegetation cover than the NDVI	Gitelson, 2004
Biochemical parameters			
**MCARI** (Modified Chlorophyll Absorption in Reflectance Vegetation Index)	1.2×(2.5×(*ρ*_*nir*_−*ρ*_*red*_)−1.3×(*ρ*_*nir*_−*ρ*_*green*_)	Capable of responding to changes in chlorophyll and estimating the chlorophyll uptake	Daughtry et al., 2000
**RDVI** (Renormalized Difference Vegetation Index)	$\frac{\rho_{nir}-\rho_{red}}{\sqrt{\left(\rho_{nir}+\rho_{red}\right)}}$	Capable of quantifying changes in a wide range of chemicals in vegetation; can be applied to a wide range of values of the leaf area index	Roujean and Breon, 1995

These 12 vegetation indices data also needed to be filtered using the Backward Feature Selection (BFS) method with the Bayesian Information Criterion (BIC) (Burnham and Anderson, 2002) as the criterion for removing redundant features before they could be used as inputs to the tiller density estimation model. The BIC was calculated as


(1) BIC=kln(n)−2ln(L)

where k is the number of model parameters, n is the number of samples, and L is the likelihood function. The BIC criterion is frequently employed as an evaluation criterion for model selection and can effectively circumvent issues that result from models being too complex due to their high accuracy. The BIC criterion also successfully prevents the selection of too many variables when there are too many dimensions and not enough samples. As a result, the minimum BIC value principle—which states that the fewest features carry the greatest information—is applied when choosing variables. In this case, based on the criterion function, the feature selection process determined the amount of tiller density information contained from the complete set of vegetation index samples; the redundant vegetation indices were then eliminated one at a time until the final subset of vegetation indices containing the necessary number of features was obtained. The selected vegetation indices were then used as inputs for training the tiller density estimation model.

The tiller density was extracted from the digital images of winter wheat gathered at Xiaotangshan that were described in **Table 1**. Each image consisted of measurements of the tiller density together with coordinate data. The images were first filtered to remove any blurred images; a total of 2600 JPG images were saved in a 1024 × 1024 × 3 RGB format. The remaining 2400 images were cutted and cropped to an 8:1:1 ratio for later use in training and validation. For transfer learning, PyTorch Hub’s DenseNet pre-training model (https://pytorch.org/hub/) was used. For this, the batch size was set to 8 and the learning rate was initially set to 0.01; Adam was chosen as the optimizer, the L2 regularization coefficient was set to 0.00005, the exponential decay rate of first-order moment estimation was set to float between 0.9 and 0.99, and the exponential decay rate of second-order moment estimation is set to 0.999. The tiller density extraction model of digital photos is finally obtained by monitoring MRE for 5 consecutive training rounds without further decline to set Early Stopping to prevent model overfitting. Then using the model to extract the tiller density for subsequent labeling of the relationship between vegetation index and tiller density.

### 2.2 Method for estimating the tiller density of winter wheat

The filtered vegetation index features were used as the input of the machine learning model. Five classical models were chosen for the machine learning method: Ordinary Least Squares (OLS), Support Vector Machine (SVM), Random Forest (RF), Gradient Boosting Regression Tree (GBRT), and Extreme Gradient Boosting (XGBoost). The samples of observed tiller density (the black points shown in **Figure 1B**) were randomly separated into training and verification sets in the ratio 8:2 for five-fold cross-validation; the labels consisted either of manually measured values of the tiller density or values that had been extracted from the digital photographs. The hyperparameters of the five models, including n estimators, max depth, min samples split, min samples leaf, and max features, were inputted in dictionary form. And the GridSearchCV method was used to adjust the hyperparameters before the optimal hyperparameter values were output. The best model was selected that gave the highest accuracy when applied to the verification set. The correlation coefficient, r, and mean relative error (MRE) were used to determine an evaluation index that described the accuracy of the tiller density estimation model. The p-value was also used as a measure of the accuracy, and only models with p< 0.05 were selected. This helped to guarantee that the results were statistically significant and could minimize overfitting caused by the small sample numbers. The correlation coefficient and MRE were calculated as follows:


(2) $$r=\sqrt{1-\frac{\sum^{}{\left(y_{i}-\bar{y_{i}}\right)}^{2}}{\sum^{}{\left(y_{i}-\tilde{y_{i}}\right)}^{2}}}$$

(3) $$MRE=\frac{1}{m}\sum_{i}^{m}\left|\frac{\bar{y_{i}}-y_{i}}{y_{i}}\times 100\%\right|$$

(4) $$t=\frac{r\sqrt{m-2}}{\sqrt{1-r^{2}}}$$

Here *y*_*i*_ is the predicted value, $\bar{y_{i}}$ is the true value, and m is the number of samples, *t* is the t-distribution; the p-value was obtained from the t-distribution corresponding to the correlation coefficient. The correlation coefficient was used to determine the model fitting regression effect: the closer the value of this was to 1, the better the regression effect. The MRE is defined as the average ratio of the absolute error of the measurement to the actual measurement. the smaller the value of this, the better the model. The p-value is a measure of the probability and gives the likelihood of an event occurring: generally p< 0.05 means a statistical difference, p< 0.01 is a statistically significant difference, and p< 0.001 is an extremely significant difference.

## 3 Results and discussion

### 3.1 Results of tiller density estimation under different experimental conditions

#### 3.1.1 Tiller density values obtained by different machine learning methods

**Figure 2:**
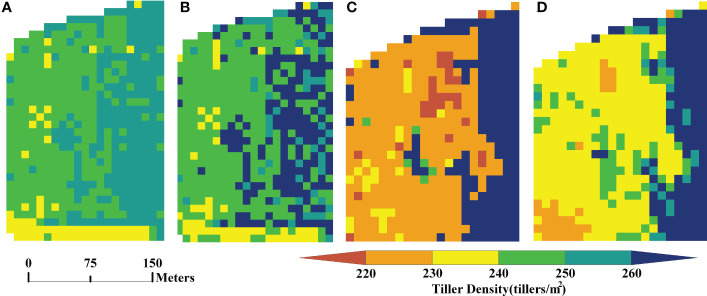
Spatial distribution of tiller density estimated using hyperspectral and multispectral data: **(A)** results for RF model applied to hyperspectral data, **(B)** results for GBRT model applied to hyperspectral data, **(C)** results for RF model applied to multispectral data, and **(D)** results for GBRT model applied to multispectral data.

Based on the UAV hyperspectral data and the Sentinel multispectral data from the same site in Xiaotangshan (marked as the blue box in **Figure 1B**), the vegetation indices listed in **Table 2** were calculated and then filtered. After filtering, the indices MCARI, RDVI, and WDRVI were obtained from the hyperspectral data, and NDVI, DVI, MCARI, MSR, RGD, RVI, and WDRVI were obtained from the multispectral data. Estimation models were then built using different machine learning models based on the manually measured values of the tiller density. Predictions of the tiller density for the same area were then made, and the spatial distribution of these values was obtained, as shown in **Figure 2** (The results for the RF and GBRT methods are shown here; the hyperspectral data were resampled to 10 m using the nearest neighbor method to facilitate comparison with the multispectral data.). The estimation results of the hyperspectral image are more compatible with the actual spatial distribution of tiller density than those of multispectral images for the various types of remote sensing data. The estimation results of GBRT are more compatible with the actual spatial distribution of tiller density for the same type of remote sensing data as those of RF (**Figure 2**).

**Figure 3:**
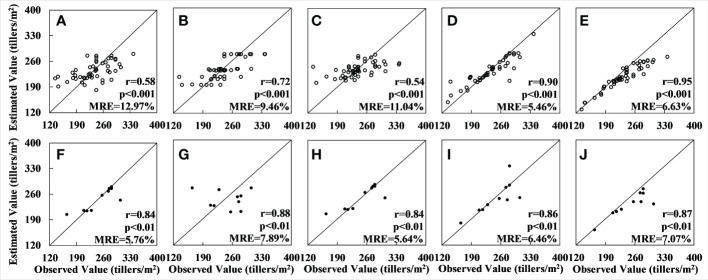
Accuracy of tiller density estimates based on the UAV hyperspectral data: **(A–E)** show the regression results for applying, respectively, the OLS, RF, SVM, GBRT, and XGBoost models to the training set; **(F–J)** show the regression results for the applying the same five models to the validation set.

Next, the verification dataset was used to verify the accuracy of the tiller density estimation. The results for the GBRT model were found to have the highest accuracy among the results for the hyperspectral data (r = 0.90 and MRE = 5.46% for the training set (see **Figure 3D**) and r = 0.86 and MRE = 6.46% for the verification set) (see **Figure 3I**). The results for the XGBoost model (see **Figures 3E, J**) had the greatest relative error up to 0.03 compared to those for the GBRT model, and the correlation coefficient for the training set was lower than the GBRT model. A comprehensive analysis also showed that the fitting effect was inferior to that for GBRT. The RF (see **Figures 3B, G**), SVM (see **Figures 3C, H**), and OLS (see **Figures 3A, F**) models performed much worse on the training set than the GBRT. The results for the RF model showed significant overfitting when the sample numbers were minimal because this model uses the average value at the root node as the outcome (see **Figures 3B, G**).

**Figure 4:**
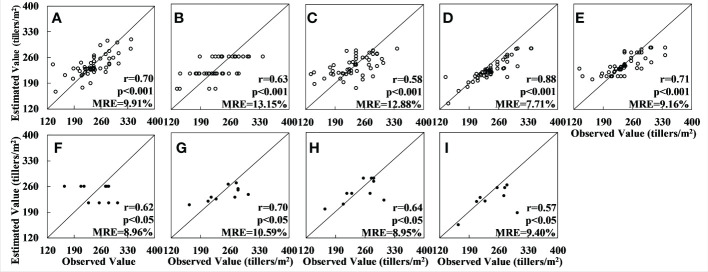
Accuracy of tiller density estimates based on Sentinel-2 data: **(A–E)** are the regression results obtained by applying, respectively, the OLS, RF, SVM, GBRT, and XGBoost models to the training set; **(F–I)** are the results obtained by applying, respectively, the RF, SVM, GBRT, and XGBoost models to the validation set. (The results for the OLS model could not be fitted and no results for the accuracy were obtained.).

Among the results for the multispectral data, the results of the GBRT model had the highest accuracy (r = 0.88 and MRE = 7.71% for the training set and r = 0.64 and MRE = 8.95% for the verification set). The XGBoost model results were poorer than those for the GBRT, with a relative error of 0.01–0.015, a lower r-value, and an inferior fitting effect, and the accuracy of the RF, SVM and OLS models was significantly lower than that of the GBRT. In particular, although the OLS method produced results with good accuracy for the training set (r = 0.70, MRE = 9.91%), validation with the verification set produced results that deviated greatly from the observed value. The fitting effect was also very poor, and serious overfitting occurred; the scatter plot for the verification set is therefore not shown in **Figure 4**. The results for the RF method also showed serious overfitting (see **Figures 4B, F**).

#### 3.1.2 Tiller density estimates based on different sample numbers

**Figure 5:**
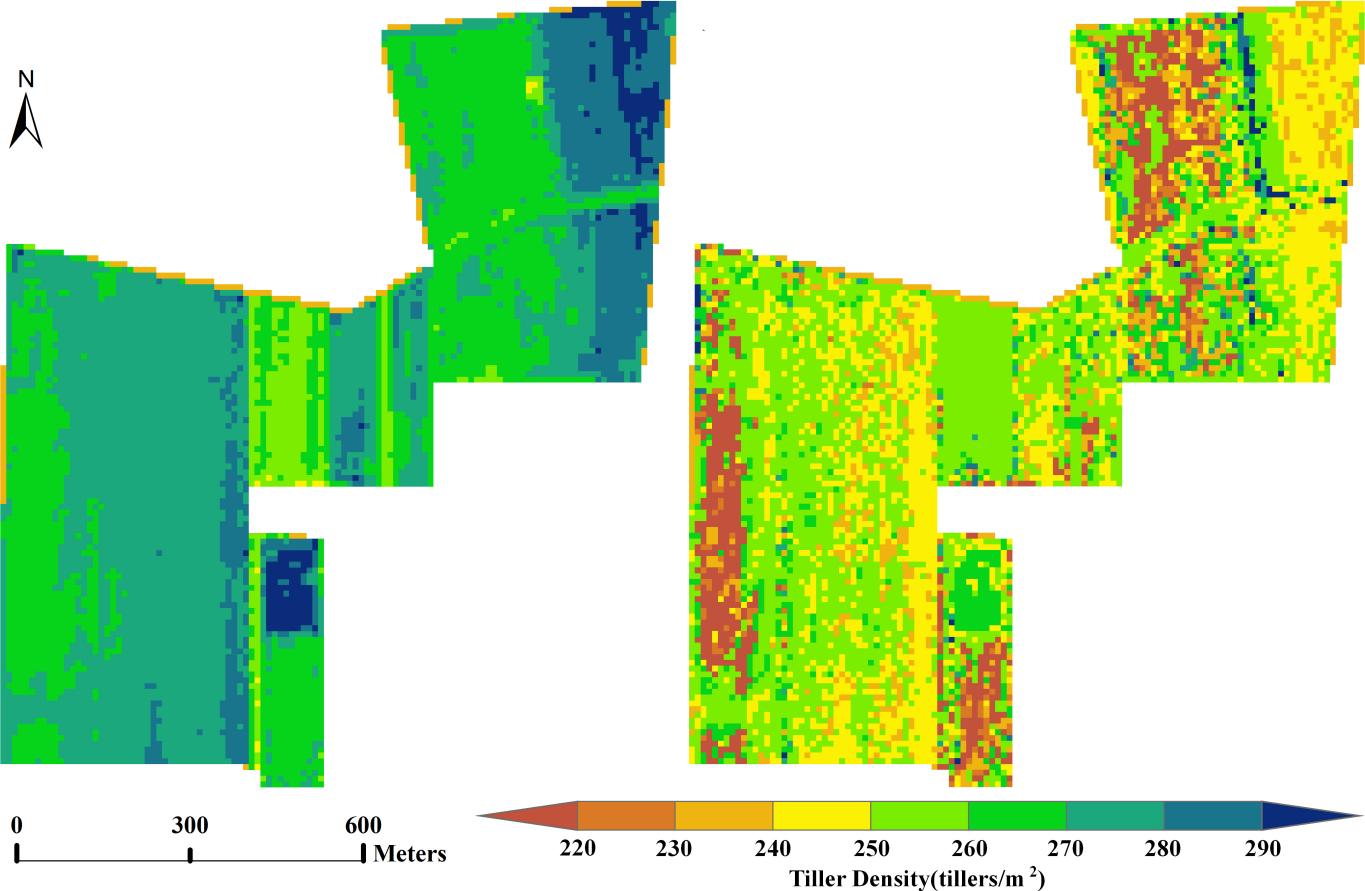
Images showing the spatial distribution of tiller density estimates obtained using Sentinel multispectral data of the whole Xiaotangshan wheat growing area: **(A)** RF model results, and **(B)** GBRT model results.

The experimental area was then expanded to include the whole of the wheat crop area at Xiaotangshan base shown in **Figure 1B**. Based on the Sentinel multispectral data, values of the vegetation indices were again calculated and filtered. The selected vegetation indices were the NDVI, DVI, MCARI, MSR, RGD, RVI, and WDRVI. Tiller density estimation models based on different machine learning models were then built. The spatial distribution of the tiller density was again obtained using these models. **Figure 5** shows the results obtained using the RF and GBRT models.

In terms of the spatial distribution, the results obtained using the RF model are more uniform than the other four models and correspond better to the actual situation. The GBRT model results are more random; there are also large differences between neighboring tiller density values in the same region.

**Figure 6:**
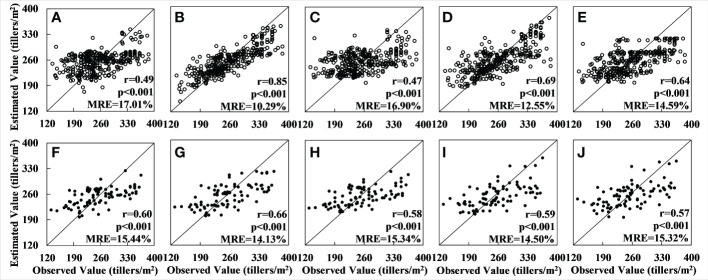
Accuracy of tiller density estimates based on Sentinel-2 data for the whole Xiaotangshan study area: **(A–E)** are the regression results for, respectively, the models OLS, RF, SVM, GBRT, and XGBoost for the training set; **(F–I)** are the regression results for the same five models for the validation set.

The results for the model accuracy obtained using the verification dataset are shown in **Figure 6**. It can be seen that, in this case, the model with the best accuracy is the RF model: for the training set the results are r = 0.85 and MRE = 10.25%, and for the verification set they are r = 0.66 and MRE = 14.13%. Among the other four models, the GBRT model performed slightly worse than the RF model on both the training and verification sets. The relative errors for the other three models – OLS, SVM, and XGBoost – have increased as a result of the larger number of samples, and the values of the accuracy are significantly lower than for the RF model. (The total number of samples, in this case, was 400; these were divided into training and verification sets using a ratio of 8:2). The experimental area was also larger.

**Figure 7:**
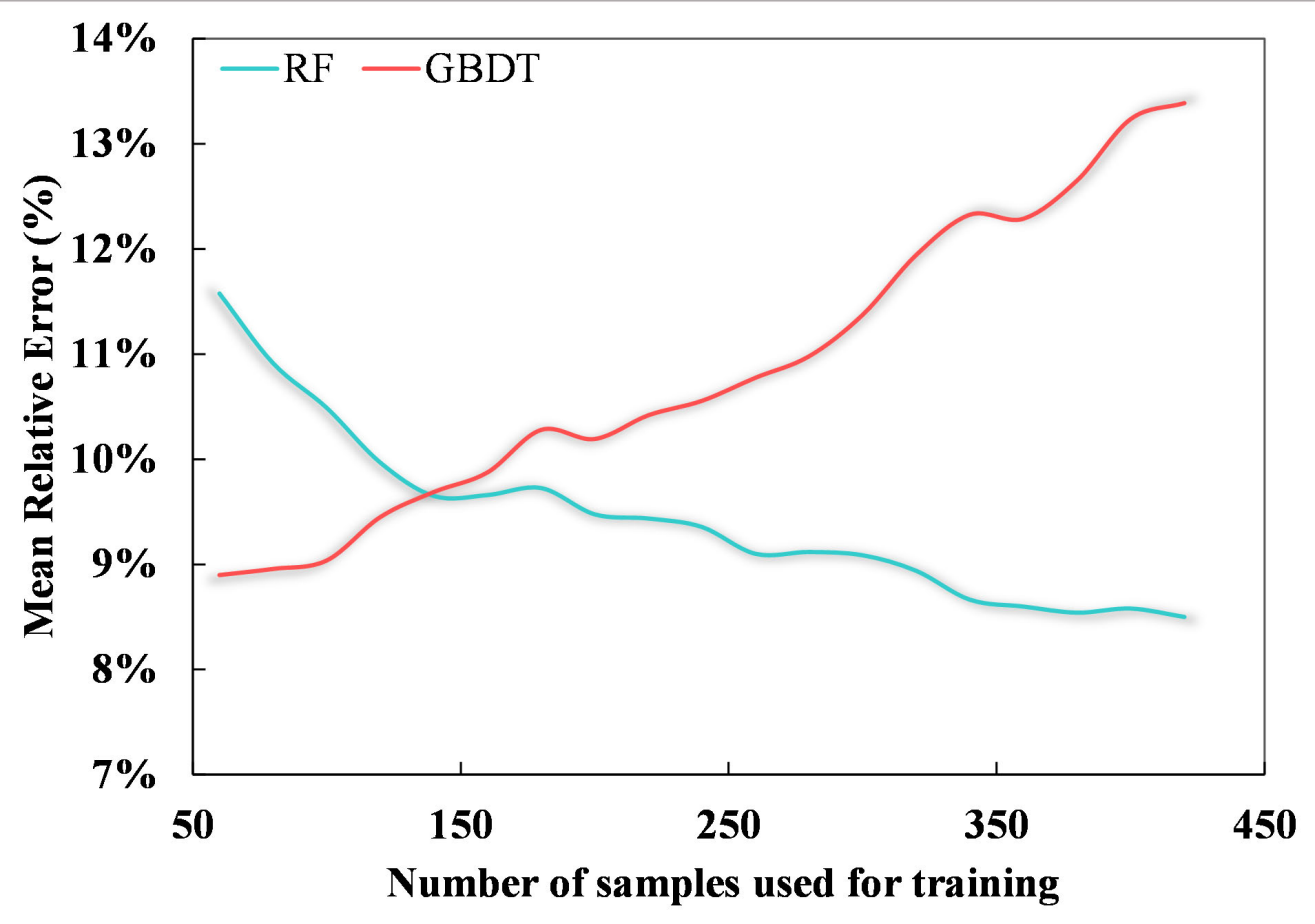
The variation in the mean relative error of RF (cyan) and GBRT (red) estimation results with increasing sample size on the validation set.

The results show that, for both the hyperspectral and multispectral datasets, when the number of data is less than 140 or so, models which are based on the boosting concept, such as GBRT and XGBoost, work best. Models that are based on the bagging concept, such as RF, perform less well due to the influence of outliers, as this leads to a concentration of values in the results. The SVM model, which maps the data from linear to nonlinear using kernel functions, is also affected by this problem to some extent. The OLS model is completely unsuitable for nonlinear fitting with a large number of features. If the sample number is greater than 140 or so, the RF model outperforms the GBRT model in terms of estimation accuracy because the RF model is sensitive to excessively unstable conditions when the sample numbers are small and cannot effectively reject outliers, resulting in overfitting. In contrast, the serial structure of the GBRT model avoids this situation when the sample numbers are small. When the number of samples increases to more than 140 or so, the RF model performs better due to good noise immunity (see **Figure 7**).

#### 3.1.3 Tiller density estimates based on different types of samples

In the next experiment, the whole wheat planting area at the Xiaotangshan base was again used. This time, however, the tiller density values were extracted from the digital photos and manually counted data. For the manually counted samples, the vegetation indices MCARI, EVI, RDVI, OSAVI, and DVI were used; the MTVI, RGD, EVI, RDVI, and OSAVI were used for the digital photographs. In Section 3.1.2, it was shown that the RF model is the most accurate when the sample number was greater than 140 or so. Therefore, a tiller density estimation model based on the RF model was built and then validated using the independent validation set (the pink points in **Figure 1B**). Based on the manually counted values, a value of r = 0.80 was obtained with a relative error of 8.66%; for the values extracted from the digital photographs, the value of r was 0.85 and the relative error was 8.98%. In both cases, p< 0.001, meaning that the results were statistically significant.

As described in Section 3.1.2, if more than around 140 samples were used, the accuracy of the RF model increased. Therefore, the independent validation set was used to validate the tiller density estimation model based on the RF model. The results for both the manually counted values and the digital photograph values were statistically significant (p< 0.001); the relative errors were 8.66% and 8.98%, respectively.

It can be concluded that tiller density extracted from digital photographs can be used in place of manually counted values as the accuracy of the estimates based on the two sets of data was similar. This would increase the effectiveness of sample collection and reduce errors due to subjective human judgment.

### 3.2 Analysis of the results obtained by applying the model to a larger area

In this section, the random forest tiller density estimation model established in Section 3.1.2 utilizing plot-scale sample data from the Xiaotangshan study site was extended to the Xiongan winter wheat crop area for use, and the same seven vegetation indices NDVI, DVI, MCARI, MSR, RGD, RVI, and WDRVI were used to predict the winter wheat tiller density in Xiongan.

**Figure 8:**
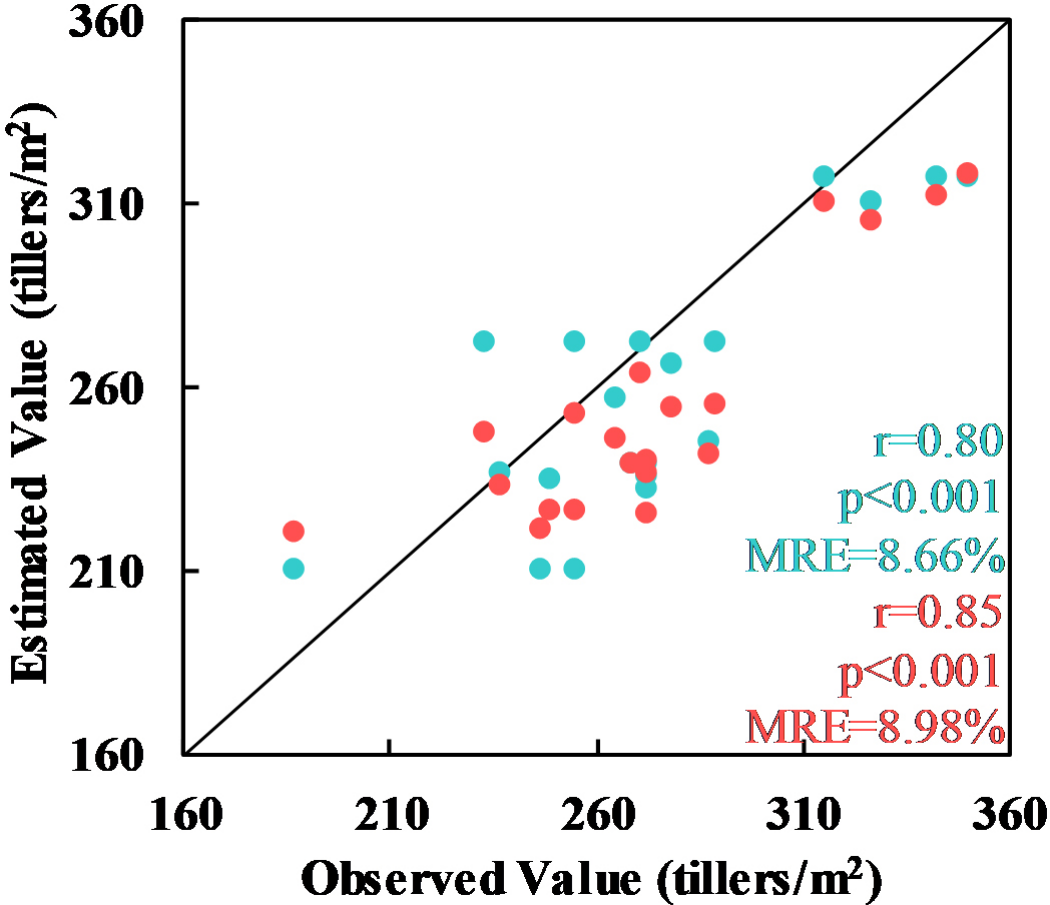
Estimated values of the tiller density based on values extracted from manually counted data (cyan) and digital photographs (red).

**Figure 9:**
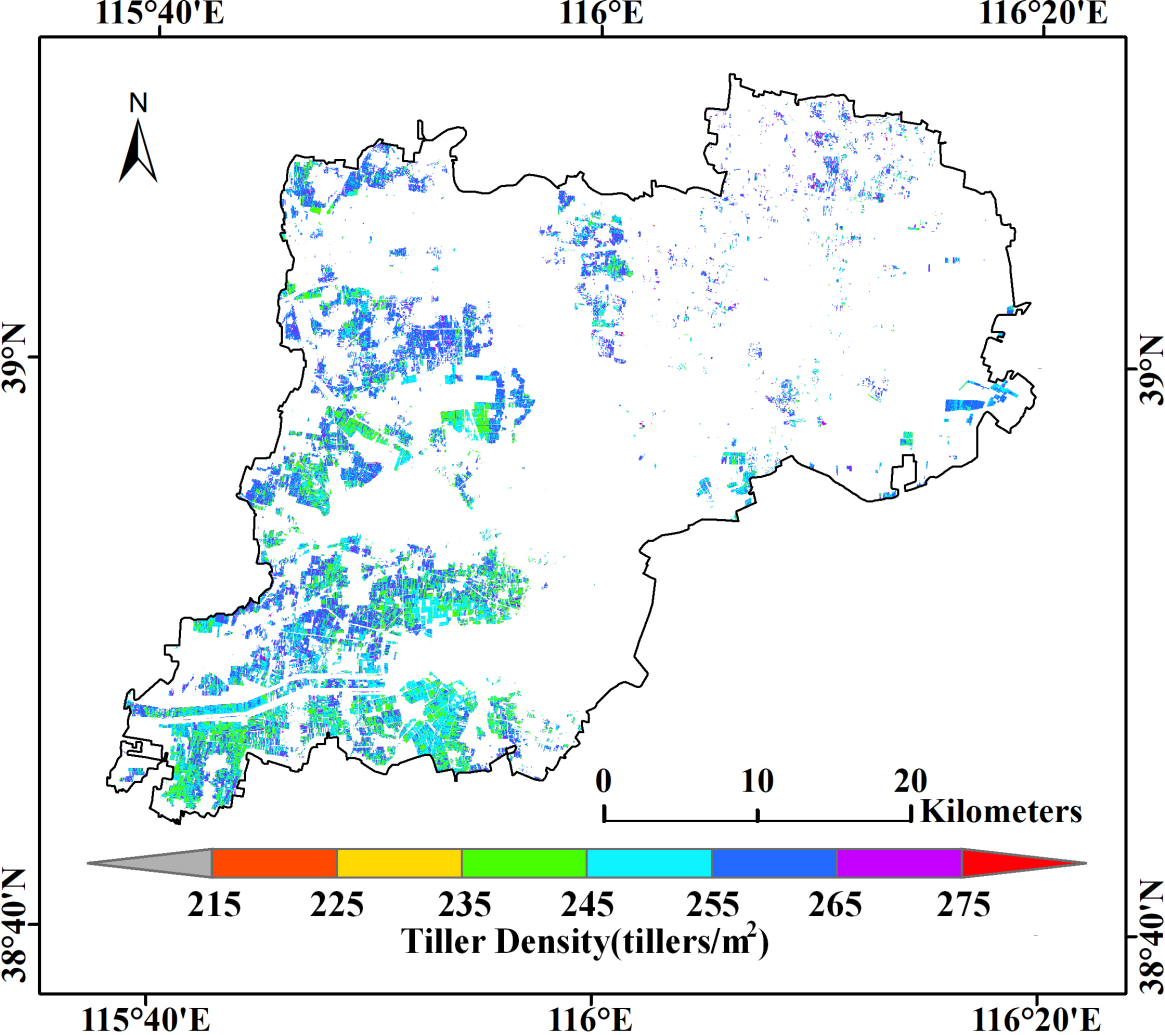
Estimated values of the tiller density obtained by extending the RF model to the Xiongan winter wheat crop area.

The estimated tiller densities obtained in this way are shown in **Figure 8**. These results distinguish better between different densities than the other four models, and the corresponding tiller densities within the same plot of land are more uniform. Even the boundaries been plots can be approximately identified, which may be because uniform sowing is used for planting in large fields. Most of the estimated values are in the range of 235–275 tillers/m2; values of 240–270 tillers/m2 correspond to about 160,000–180,000 tillers per acre, which is in agreement with the 120,000–180,000 tillers per acre used when sowing (see **Figure 9**).

**Figure 10:**
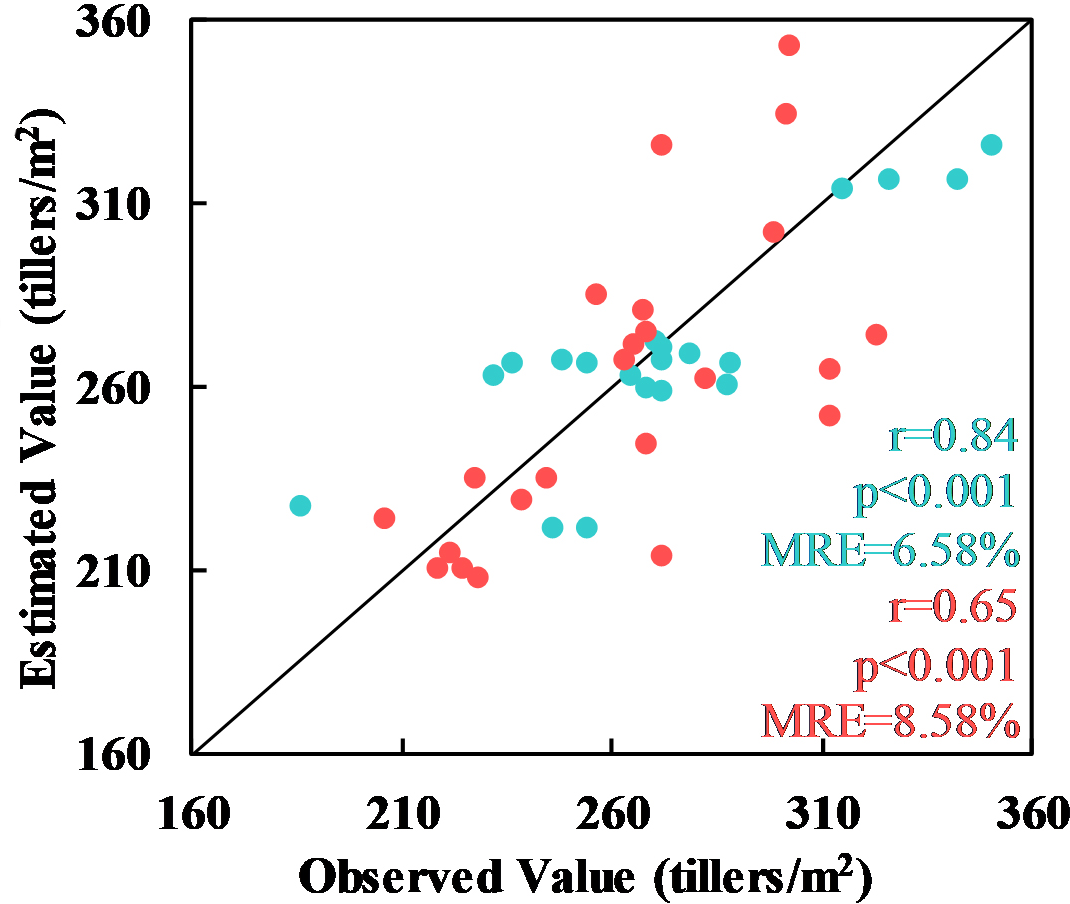
Comparison of the tiller density estimates at Xiaotangshan (cyan) and in the Xiongan study area (red) with another validation dataset.

Validation of the Xiongan results using the ground validation points (the pink points in **Figure 1A**) gave a statistically significant value of r of 0.65 and a relative error of 8.58% with p< 0.001. The same model was also validated as statistically significant using other validation points (the pink points shown in **Figure 1B**) at the Xiaotangshan research site (r = 0.84, MRE = 6.58%, p< 0.001) (see **Figure 10**).

In conclusion, the validation results of both spatial distribution and ground observed values two ways, demonstrate that the plot-scale tiller density estimation model obtained using data from the Xiaotangshan study site can be extended to the county scale. Although the validation of the results showed that the accuracy of the Xiongan results was lower than that of the Xiaotangshan results, the relative error was still less than 10%, which is sufficient for making estimates of the tiller density of winter what over large areas based on satellite remote sensing data.

### 3.3 Comparison and analysis with other tiller density estimation methods

The tiller density estimation method developed in this study was primarily based on the spectral features of remote sensing data, and quantitative estimates that benefitted from the advantages of machine learning methods as applied to nonlinear regression were obtained using multiple vegetation indices after feature selection. In contrast, the traditional approach to tiller density estimation is generally based on the high degree of correlation between NIR bands and the tiller density and uses regression based on a single vegetation index (Flowers et al., 2001; Flowers et al., 2003). The results obtained in this way are often biased (relative error > 20%) (Scotford and Miller, 2004), and the generalizability of the method is poor due to the limited applicability of the chosen vegetation index. For example, the NDVI does not fully reflect the wheat tiller density in situations where the leaf area index is particularly high or low or where the amount of cover is high; the sensitivity of the RVI decreases significantly when the vegetation cover is below 50% and thus does not fully reflect the number of wheat tillers (Wu et al., 2022). To address these problems, in this study, multiple vegetation indices were used to complement each other to meet the accuracy requirements of precision agriculture.

Whether based on 2D RGB images or 3D point cloud data, the results of tiller density estimation based on an image segmentation model are susceptible to the influence of the wind as well as the lighting conditions (Roth et al., 2020). The resolution of the 2D RGB images also needs to be high (Jin et al., 2017). Both types of data are mainly captured by UAVs or handheld cameras. This can lead to errors associated with the chosen sampling location selection as a result of spatial heterogeneity (Liu et al., 2017), meaning that the acquired data can only be applied at the scale of an individual plot of land.

It has been shown that our method meets the accuracy requirements to estimate tiller density using high-resolution remote sensing data and can be used to obtain complete maps of the spatial distribution of the tiller density within an individual plot, which is something that methods based on image segmentation cannot do. It has also been shown that the proposed method can be extended to larger-scale tiller density estimation and monitoring, thus taking full advantage of the ability of remote sensing to quickly provide data covering large areas and potentially aiding more accurate fertilizer application and yield estimation.

In addition, this study has also provided a preliminary demonstration of the feasibility of using digital photographs instead of manual counting. In the future, the use of accurate values of the tiller density extracted from digital photographs could be extended to larger samples to achieve low-cost estimates of the spatial distribution of the tiller density at large spatial scales, something which has not been considered in previous studies.

### 3.4 Analysis of factors affecting the accuracy of the tiller density estimation

#### 3.4.1 Relationship between the tiller density and the type of remote sensing data

According to the results presented in Section 3.1.1, for all five machine learning methods that were tested, the tiller density estimates based on the hyperspectral data were more accurate than those based on the multispectral data. This was primarily due to the hyperspectral data’s high spectral resolution and the large number of bands, which allowed vegetation indices to be constructed using the bands that were correlated most strongly with the tiller density. Other vegetation indices could be used as well as the narrow-band indices that were used in this study (Borengasser et al., 2007). However, the central wavelengths of the bands of the Sentinel data were marginally less well correlated with the tiller density than the hyperspectral data were, which led to the lower accuracy of the results for the multispectral data.

#### 3.4.2 Relationship between the tiller density and vegetation indexes

As described in Section 3.1, the features selected from hyperspectral data were the MCARI, RDVI, and WDRVI, the features selected from the Sentinel data were the NDVI, DVI, MCARI, MSR, RGD, SR, and WDRVI, the features selected from the manual measurements were the MCARI, EVI, RDVI, OSAVI, and DVI, and the features selected from the values extracted from the digital photographs were the MTVI, RGD, EVI, RDVI, and OSAVI. These vegetation indices are mainly related to vegetation parameters such as chlorophyll content, leaf area index, vegetation cover, and aboveground biomass. The vegetation indices related to the leaf area index and vegetation cover are the most frequent, which is a crucial measure of crop growth (Xing et al., 2021). The strong correlation between the vegetation indices that reflect the chlorophyll content of the wheat canopy surface (such as the NDVI) and the tiller density has been demonstrated in previous studies, (Flowers et al., 2001; Flowers et al., 2003). This is reflected in how the tiller density affects the value of the leaf area index and the canopy density (Bates et al., 2021). Changes in the canopy density also cause changes in the vegetation cover, which means that the tiller density can be estimated from the vegetation cover: this is consistent with the observation of a strong correlation between the vegetation cover and plant density in wheat. (Wang et al., 2020; Wu et al., 2022).

Although these vegetation indices may be linearly correlated with each other, it is still possible to estimate the tiller density from them thanks to the ability of machine learning to handle covariance problems (Liang et al., 2015). Therefore, consideration should be given to the use of vegetation indices related to vegetation cover when selecting which indices to use for estimating tiller density.

#### 3.4.3 Relationship between tiller density and texture

Texture, another important class of features that can be used for the inversion of vegetation parameters, is widely used with machine learning inversion methods such as AGB (Yue et al., 2019). In this study, based on the grayscale coevolution matrix (GLCM), we also attempted to calculate eight image texture features for four different bands: mean, variance, homogeneity, contrast, dissimilarity, entropy, second-order moments, and correlation (Yue et al., 2019). Together with the vegetation index, these features were filtered based on the BIC criterion using the BFS method; it was found that the vegetation index contained the most information about the tiller density while the image texture features contained little information. For both the UAV and Sentinel-2 data, the EVI and the other vegetation indices that made use of the NIR bands ranked highly in terms of tiller density information content, which is consistent with the findings of Flowers et al. (Flowers et al., 2001; Flowers et al., 2003; Scotford and Miller, 2004). The reason for this may be that the tiller density at tillering stage is a relatively microscopic feature: the individual tillers overlap each other, which makes them difficult to distinguish with the naked eye, and an extremely high spatial resolution (e.g., 0.02 cm) is required to extract information using machine vision methods (Liu et al., 2018). The resolution of the data used in this study did not meet this requirement. However, as the tiller density increases, the canopy density and the amount of cover change, which also affects the spectral features (the reflectance in the near-infrared band increases). The vegetation indices can amplify this effect, thus making more tiller density information available and better estimates possible.

## 4 Conclusion

In this study, we attempted to estimate the tiller density of winter wheat at the tillering stage based on a combination of multiple remotely sensed vegetation indices and using machine learning models.

(1) Under all experimental conditions, the relative error in the estimates of the tiller density was in the range of 5.46%–12.97% for the hyperspectral data and 7.71%–13.15% for the multispectral data. The estimates based on the hyperspectral data were thus more accurate, and in both cases, the relative error was less than 10%, which is the usual level of accuracy required.
Based on the results of this study, tiller density can be extracted from digital images instead of by manual counting during ground sampling as the results for the tiller density obtained in this way were just as accurate as those based on the manual method.
(2) The application of this machine learning model for estimating the tiller density of winter wheat based on plot-scale samples could be extended to the county scale and still meet the requirement of having a relative error of less than 10% although the results may be affected by the spatial heterogeneity of the wheat.
Among the different methods that were tested, the random forest and gradient boosting tree methods gave the most accurate results. The gradient boosting tree is most suitable for sample numbers less than around 140; the random forest is suitable for sample numbers greater than around 140 or with outliers.
(3) Vegetation indices associated with the vegetation cover and leaf area index are suitable for use as features for estimating the winter wheat tiller density. The texture features in remote sensing imagery contain almost no information on the winter wheat tiller density and are hence not a suitable basis for making estimates of the tiller density.

## Data availability statement

The original contributions presented in the study are included in the article/supplementary material. Further inquiries can be directed to the corresponding author.

## Author contributions

The experiment was mainly conceived and designed by BZ. JH, RY, YL, CX, CL, TD, MF, HY, WH, BL, EC, and SY performed the experiments. BZ, JH, and DP analyzed the data. The algorithm development was mainly accomplished by BZ, JH, and DP. JH wrote the manuscript and BZ made very significant revisions. DP and MW also read and improved the final manuscript. All authors contributed to the article and approved the submitted version.
